# The power of pooled analysis to inform optimal dosing strategies for Artemisinin Combination Therapies (ACTs)

**DOI:** 10.1186/1475-2875-11-S1-P40

**Published:** 2012-10-15

**Authors:** Philippe J Guerin, Clarissa Moreira, Prabin Dahal, Patrice Piola, Kasia Stepniewska, Ric Price

**Affiliations:** 1 WorldWide Antimalarial Resistance Network (WWARN), Oxford, UK; 2 Centre for Tropical Medicine, Nuffield Department of Clinical Medicine, University of Oxford, Oxford, UK; 3 Menzies School of Health Research, Darwin, Australia

## Background

Antimalarial efficacy is dependent on administration of a curative dose determined by the pharmacokinetic and dynamic profile of the drug and the age and weight of the patient. Optimal dosing strategies are frequently compromised by pragmatic constraints resulting in patients receiving a wide range of mg/kg dose. WWARN has established a scientific research project aiming at pooling all relevant patient level data from ACTs studies conducted in malaria endemic areas. This study aimed at investigating the consequences of variance in dosing strategies of key ACTs and their effect on clinical efficacy.

## Methods

A systematic review generated a list of all antimalarial clinical studies since 1960. Studies relevant to specific pooled analyses were identified and principal investigators approached to contribute individual patient data, which were then compiled into a standard format according to a transparent Data Management and Statistical Analysis Plan [1]. Data were collated for artemether-lumefantrine (AL), artesunate-amodiaquine (AS+AQ) and dihydroartemisinin-piperaquine (DHA+PQP) and analysed separately according to an a priori analytical plan to identify key risk factors for treatment efficacy, recrudescence and new infection by day 28. Univariate and multivariate risk factors were identified using Cox’s regression model with frailty shared across the studies to adjust for the differences between studies. Optimal mg/ kg dosage of partner drugs which best predicted the PCR adjusted recrudescence were explored using logrank statistics for pre-defined weight/age categories.

## Results

The WWARN repository currently contains over 75,000 individual patient records, 54% of which were treated with ACTs. In the current pooled analysis there are 10,913 patients treated with AL, 6,073 with AS+AQ and 4,739 with DHA+PQP, constituting 45%, 40% and 35% of the entire published data for these three drugs respectively. For AL, significant multivariate risk factors for recrudescence were baseline parasitaemia (log-scale) [AHR: 1.11, 95% CI: 1.00-1.23] and low weight category of 5-14 kg [AHR: 2.08, 95% CI: 1.06-4.11]. However, the mg/ kg dosage of lumefantrine was found not to be associated with the recrudescent failures [P=0.83] in the final model. Patient treated with non-fixed combination of AS+AQ were at 2.8 fold [95% CI: 1.53-5.29] greater risk of recrudescent failure compared to those treated with fixed dose combination (FDC). For the non-fixed combination of AS+AQ, logged baseline parasitaemia [AHR: 1.24, 95% CI: 1.07-1.45], low age category [age 1-5 years (AHR: 3.23, 95% CI: 0.90-11.94)] and the mg/kg amodiaquine dose [AHR: 0.96, 95% CI: 0.93-1.01] were the major risk factors for recrudescence in the final multivariate model. No risk factors were significantly associated with failure with the FDC. For DHA+PQP, patients in age group <12 years [AHR: 4.13, 95% CI: 1.06-15.94] and patients receiving an overall piperaquine dose of <48 mg/ kg [AHR: 1.69, 95% CI: 0.95-3.02] were at greater risk of recrudescence.

## Conclusions

Pooled analyses of diverse clinical studies are feasible using standard algorithms and semi-automated processing and can provide new insights on risk factors of failure. Large standardised datasets provide substantial power to explore the impact of different dosing strategies and derive optimal treatment protocols.
